# Does Tumour Contrast Retention on CT Immediately Post Chemoembolization Predict Tumour Metabolic Response on FDG-PET in Patients with Hepatic Metastases from Colorectal Cancer?

**DOI:** 10.1155/2019/7279163

**Published:** 2019-11-04

**Authors:** K. T. Tan, R. Rakheja, C. Plewes, P. Mondal, H. Lim, S. Ahmed, E. Lee, R. Otani, Y. Luo, J. Shaw

**Affiliations:** ^1^Department of Medical Imaging, University of Saskatchewan, 103 Hospital Drive, Room 1566, Saskatoon, SK, Canada S7N 0W8; ^2^Department of Medical Imaging, Schulich Medicine & Dentistry, University of Western Ontario, London Health Sciences Centre, University Hospital, London, ON, Canada N6A 5A5; ^3^Clinical Research Support Unit, College of Medicine, University of Saskatchewan, 107 Wiggins Road, Saskatoon, SK, Canada S7N 5E5; ^4^Department of Community Health & Epidemiology, University of Saskatchewan, 107 Wiggins Road, Saskatoon, SK, Canada S7N 5E5; ^5^Saskatoon Cancer Centre, 20 Campus Drive, Saskatoon, SK, Canada S7N 4H4; ^6^Department of Surgery, University of Saskatchewan, 103 Hospital Drive, Saskatoon, SK, Canada S7N 0W8

## Abstract

**Purpose:**

The exact mechanism of action of chemoembolization with drug eluting beads loaded with irinotecan (DEBIRI) in colorectal cancer is undetermined. Posttreatment tumour contrast retention often seen on CT immediately post procedure is of indeterminate significance. This study is aimed at assessing if metabolic response on PET-CT can be related to posttreatment tumour contrast retention.

**Materials and Methods:**

In this retrospective study, a total of 17 patients with a total of 55 marker lesions were recruited.

**Results:**

The area of tumour contrast retention can be matched to a hypometabolic area on subsequent PET-CT in over 36 lesions (65.5%). Out of the 55 lesions, a total of 38 marker lesions in 11 patients who also had pre-DEBIRI PET-CT were analyzed for disease response. 10 out of 10 lesions that had a complete response on PET-CT were found to demonstrate contrast retention throughout the tumour. 12 out of 13 (92.3%) tumours that had a partial metabolic response on PET-CT were found to demonstrate contrast uptake in the hypometabolic area only. In the 15 lesions that had progression/no response, 13 (86.6%) demonstrated no relationship between tumour contrast retention and tumour response. There was a significant correlation between contrast retention and disease response (*P* < 0.001).

**Conclusion:**

Our study showed that PET-CT response can be associated with post embolization contrast retention. The data suggests blood stasis, for which tumour contrast retention is a surrogate marker, is important for the PET-CT metabolic response. The authors propose that tumour contrast retention is an important embolization endpoint in DEBIRI.

## 1. Introduction

Chemoembolization with drug eluting beads loaded with irinotecan (DEBIRI) is recognized as an adjunctive treatment for colorectal cancer with liver-dominant or liver-limited metastases [1]. At present, consensus guidelines recommend its use in patients who either are unsuitable for or have failed conventional treatment.

Indeed, the use of DEBIRI has been shown to be promising in the treatment of patients who have disease refractory to conventional therapy [2]. In addition, a small phase III study appears to demonstrate a superiority of DEBIRI over FOLFIRI in patients with metastatic colorectal cancer [3].

Irinotecan is a prodrug that requires activation by carboxylesterase into its active agent, SN38 [4]. As this mainly occurs in a healthy liver, lobar administration of DEBIRI is recommended to achieve optimal activation of the drug. This is in contradistinction to the superselective administration of doxorubicin transarterial chemoembolization for hepatocellular carcinoma.

A pharmacokinetic study in a pig model suggests that DEBIRI offers a longer exposure to irinotecan and SN-38, when compared to intravenous administration of the drug [4]. It is hypothesized that this finding is due to decreased clearance by impaired hepatic blood flow from embolization as well as decreased drug metabolism due to local delivery [4].

**Figure 1:**
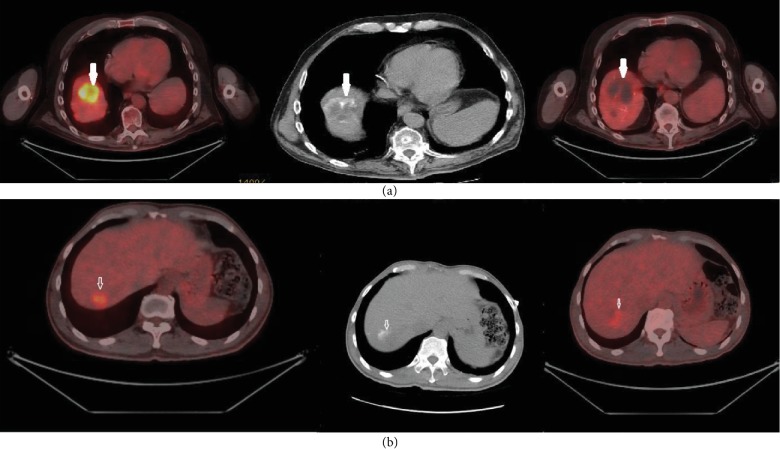
(a) The top row illustrates a Type 1 match associated with a complete response. The top left picture demonstrates a right lobe hypermetabolic lesion. There is contrast retention post-DEBIRI (middle column). There is no discernible metabolic activity in the top right picture. (b) The bottom row demonstrates a Type 1 match associated with a partial response.

Based on the above premise, it would be expected that lobar administration of DEBIRI would achieve equivalent “tumour kill” in the entire treatment field. However, a serendipitous observation on one of our patients that the area of tumour response on PET-CT appeared to correspond to the area of contrast retention post chemoembolization would suggest otherwise (Figure 1). A previous paper on bland embolization of hypervascular tumours suggests that lack of contrast retention on immediate post embolization CT is associated with a higher probability of not achieving complete response [5]. However, the presence of immediate contrast retention has not previously been shown to be associated with treatment success on “hypovascular” tumours such as colorectal cancer.

We, therefore, decided to test the hypotheses that (1) the area of tumour contrast retention corresponds with the hypometabolic area of FDG on PET-CT and (2) tumour contrast retention is associated with a metabolic response. If these were found to be true, the findings would perhaps suggest that a local embolic effect on colorectal tumour may play an important part in the mechanism of action of DEBIRI.

## 2. Materials and Methods

This retrospective study was approved by the local Research Ethics Board.

### 2.1. Subjects

As part of routine clinical care, all patients at our institution had a nonenhanced CT scan immediately post DEBIRI. All patients were discussed at the local tumour board prior to DEBIRI. Records of all patients who underwent DEBIRI for colorectal cancer between January 2013 and December 2017 were reviewed.

All patients who had PET-CT scans between 1 and 3 months after the completion of DEBIRI were included. A total of 17 patients (3 women and 14 men) and 55 marker lesions were included. Patient ages ranged from 42 to 85 years (mean ± SD: 63.4 ± 11.1).

Out of the 17 patients, data from 11 patients who had a PET-CT scan prior to the DEBIRI were used for additional analysis to assess for tumour response. A total of 38 marker lesions were included for analysis. Patient ages ranged from 42 to 68years (mean ± SD: 59.1 ± 9.5). There were 9 men and 2 women.

### 2.2. Embolization

All DEBIRI were performed by either KT or RO, who are both experienced interventional radiologists. All but two DEBIRI were performed using 70-150 *μ*m DC Bead M1 (BTG, Farnham, UK). One patient was embolized with 100-300 *μ*m DC Bead (BTG, Farnham, UK), while another one was treated with DC Bead LUMI 70-150 *μ*m (BTG, Farnham, UK). DEBIRI was performed using a previously published consensus protocol [6]. Briefly, proximal catheterization of either the common hepatic or proper hepatic artery was performed using a 5Fr catheter. The relevant lobar artery was then catheterized with either a 2.4 Fr or a 2.8 Fr microcatheter. Beads loaded with 100 mg of irinotecan were administered by slow “puff” injections. Complete administration of the drug was achieved in all patients without achieving angiographic stasis.

In cases of bilobar disease, a total of 4 treatments were performed, alternating between each lobe, with an interval of 2 weeks between procedures. Unilobar disease was treated by two procedures, with a one-month interval between treatments.

### 2.3. CT and PET-CT Imaging

Immediate postprocedural CT scans, 1-3-month post-DEBIRI PET-CT, and pre-DEBIRI PET-CT were interpreted separately by two imaging specialists (KT and RR), one of whom is a nuclear medicine physician with an interest in PET-CT (RR). In cases of disagreement on the initial read, a consensus was reached by discussion.

### 2.4. Patients with a Post-DEBIRI PET-CT

**Figure 2:**
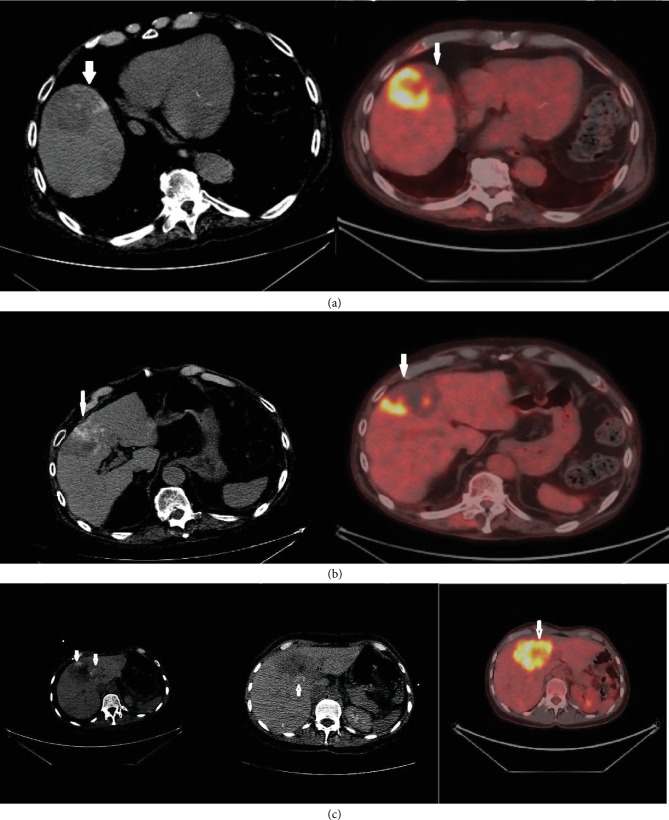
(a, b) The column on the left demonstrates post embolization contrast retention on CT (arrows). The PET-CT images on the right demonstrates corresponding hypometabolic areas. Findings are in keeping with significant overlap. (c) The CT on the left was obtained after DEBIRI treatment to the left lobe of the liver. The middle CT demonstrates contrast retention after the DEBIRI treatment to the right lobe of the liver. There is no corresponding hypometabolic area on PET-CT. Findings would be in keeping with a “no match.”

If the hypometabolic area on post-DEBIRI FDG PET corresponds to the area of post-DEBIRI contrast retention by 75-100% by visual assessment, it is considered a “significant overlap.” If there is less than 75% overlap, it is considered “no overlap” (Figure 2).

### 2.5. Patients with Both Pre- and Post-DEBIRI PET-CT

If the area of contrast retention matches the hypometabolic area in the post-DEBIRI PET-CT by 75-100%, it is considered to demonstrate a Type 1 match; a 25-75% overlap is considered a Type 2 match, while 0-25% overlap is considered a Type 3 match (Figure 1).

As a pre-DEBIRI PET-CT is available for comparison, the overall tumour response can also be determined. The metabolic response was also graded as “complete” when there is complete loss of FDG uptake and no enlargement, “partial” when there is some reduction in FDG uptake and no enlargement, and “no response/progression” when there is no reduction in FDG uptake and/or enlargement.

### 2.6. Statistical Analysis

The mean, standard deviation, and range were provided for continuous variables. We reported numbers and percentages for categorical variables. Fisher's exact test was used to find an association between categorical variables. A *P* value of <0.05 (two-sided) was considered statistically significant. Statistical analysis was performed using the SAS 9.4 software (SAS, Carry, NC, USA).

## 3. Results

### 3.1. Patients with a Post-DEBIRI PET-CT

36 (65.5%) of lesions demonstrated a significant overlap. 19 (34.5%) of lesions demonstrated no overlap (Table 1).

### 3.2. Patients with Pre- and Post-DEBIRI PET-CT

Out of the 38 lesions, 23 demonstrated a Type 1 match (60.5%), 2 had a Type 2 match (5.3%), and 13 had a Type 3 match (34.2%) (Table 1). 10 (26.3%) of patients demonstrated complete metabolic response, 13 (34.2%) demonstrated partial metabolic response, and 15 (39.5%) demonstrated no response/progression.

All 10 (100%) lesions that demonstrated complete metabolic response were associated with a Type 1 match. Contrast retention in lesions that demonstrated complete metabolic response was found to cover the entire tumour area found on the pre-DEBIRI PET-CT.

Out of the 13 lesions that demonstrated partial metabolic response, 12 (92.3%) were associated with a Type 1 match while 1 (7.7%) was associated with a Type 2 match. All 12 lesions that were associated with a Type 1 match had areas of high FDG uptake that corresponded to areas that did not demonstrate contrast retention post-DEBIRI.

In the 15 lesions that demonstrated no response/progression, 1 (6.7%) demonstrated a Type 1 match, and 1 (6.7%) demonstrated a Type 2 match, while the remaining 13 (86.6%) demonstrated a Type 3 match.

Due to the small number of lesions with a Type 2 match, this category was grouped with lesions that demonstrated a Type 3 match for formal statistical analysis (Table 2). The results are highly significant (*P* < 0.0001), with a Type 1 match highly correlated with complete and partial metabolic responses while Type 2 and Type 3 matches are associated with no response/progression.

## 4. Discussion

This study demonstrated that although tumour contrast retention post-DEBIRI does not necessarily predict response, areas that do demonstrate a metabolic response on PET-CT are associated with contrast retention immediately post procedure. Almost all lesions that demonstrated a partial metabolic response had areas that did not demonstrate contrast retention on the post-DEBIRI CT. Indeed, the areas that remained hypermetabolic in these lesions corresponded to the regions with no post-DEBIRI contrast retention.

Multiple previous papers have demonstrated that contrast retention posttransarterial chemoembolization/bland embolization can be related to tumour response in hypervascular tumours [5]. However, this has never been described in the so-called “hypovascular” tumours such as colorectal metastases.

It has been known for at least half a century that colorectal metastases in the liver are supplied predominantly by branches of the hepatic artery, and this has been exploited clinically by the use of hepatic artery infusion therapies which allow high doses of chemotherapy to be administered to liver metastases [7].

The phenomenon of contrast retention post embolization is commonly seen. It is due to the stasis/slow flow of blood in the targeted area, which prevents the “washout” of contrast. Embolic agents in the hepatic arterioles are not typically associated with hepatic parenchymal contrast retention in a colorectal DEBIRI practice due to the mixing of blood from the portal venous system.

However, the results from this study do raise the question as why response to DEBIRI, which is hypothesized to work due to a combination of local high doses of irinotecan/SN38 and decreased hepatic clearance, is associated with tumour contrast retention. The simplest explanation would be that direct ischaemic insult/impairment of tumour microvasculature has an antitumour effect. Indeed, the clinical use of antiangiogenic agents such as bevacizumab in colorectal cancer highlights the importance of tumour vasculature in this disease. However, this may be an oversimplification as there is evidence in animal models to suggest that ischaemic insult might actually promote tumour growth [8]. In addition to a direct ischaemic effect, it is possible that blood stasis may reduce the clearance of active SN38 from the tumour.

Another possible explanation behind this observation might be the accumulation of high doses of irinotecan in the area of stasis. Carboxylesterases are a diverse group of enzymes, many of which can activate irinotecan [9]. Studies suggest that carboxylesterase 2 is the primary enzyme responsible for this process at doses reached during intravenous administration of the drug [9]. However, many other carboxylesterases are capable of activating irinotecan at much higher K*m* values. It is possible that levels of irinotecan achieved during DEBIRI could optimize its activation by saturating carboxylesterase 2 and recruiting other carboxylesterase.

*β*-Glucuronidase is found in colorectal cancer tissue [10]. This enzyme promotes the reactivation of SN38 after it has been inactivated by formation of its glucuronide, SN38G. This pathway may be clinically important [10]. By impairing the removal of SN38G, due to blockage of blood flow, DEBIRI could potentially promote its reactivation.

Whatever the underlying explanation, the results are clinically important. The results suggest that adequate embolization of hepatic colorectal metastases is important to achieve adequate tumour control. However, over embolization is to be avoided as it is associated with an excessive risk of complication [6]. Based on the current results, it is not unreasonable to use either on-table cone beam CT or an immediate post embolization CT to determine tumour contrast retention. The need for more aggressive treatment of tumours (e.g., by subselective embolization) that do not demonstrate contrast retention should then be considered.

It is surprising that despite lobar administration of the embolic agent and all precautions taken to disrupt laminar flow/ensure adequate mixing of the embolic, areas of viable tumour tissue remain untreated. Unfortunately, this cannot be adequately detected by contrast injection as all our cases were performed after an angiographic examination to confirm good catheter placement. Indeed, it is possible that in situ separation of the bead suspension from the contrast column is the likely explanation for undertreated tumour. The use of radiopaque beads, such as LUMI (BTG, Farnham, UK), might overcome this issue, although further studies are required before a formal recommendation can be made.

## 5. Conclusion

To conclude, tumour contrast retention post-DEBIRI should be considered an ideal end-point. Further studies should be undertaken to elucidate how stasis of blood in a colorectal cancer metastasis will influence treatment response. It would also be interesting to correlate other surrogate markers of blood flow (e.g. perfusion) to treatment response.

## Figures and Tables

**Table 1:** Frequency distribution.

Variables	Number (percentage)
All Lesions (*n* = 55)	
Significant overlap	36 (65.5)
No overlap	19 (34.5)

Lesions with a baseline PET/CT scan (*n* = 38)	
Match type	
Type 1	23 (60.5)
Type 2	2 (5.3)
Type 3	13 (34.2)

Metabolic response	
Complete	10 (26.3)
Partial	13 (34.2)
No response/progression	15 (39.5)

**Table 2:** Association of metabolic response with response match type. Response type and match were significantly associated with metabolic response.

Variables	Metabolic response (*n* = 38)			*P* value^∗^
	Complete (*n*, %)	Partial (*n*, %)	Progression/no response (*n*, %)	
Match type				
Type 1	10 (100)	12 (92.3)	1 (6.7)	N/A
Type 2	0	1 (7.7)	1 (6.7)	
Type 3	0	0	13 (86.6)	

Match type				
Type 1	10 (100)	12 (92.3)	1 (6.7)	<0.0001
Types 2 & 3	0	1 (7.7)	14 (93.3)	

In complete response, partial response and progression/no response, percentages of type 1 match type were 100, 92.3, and 6.7, respectively.

## Data Availability

The retrospective data used to support the findings of this study are included within the article.

## Abbreviations

FDG: Fluorodeoxyglucose
PET: Positron emission tomography
CT: Computed tomography
DEBIRI: Drug eluting beads loaded with irinotecan
FOLFIRI: Folinic acid, irinotecan, and fluorouracil.

## References

[1] Yoshino T., Arnold D., Taniguchi H. (2018). Pan-Asian adapted ESMO consensus guidelines for the management of patients with metastatic colorectal cancer: a JSMO–ESMO initiative endorsed by CSCO, KACO, MOS, SSO and TOS. *Annals of Oncology*.

[2] Martin R. C. G., Joshi J., Robbins K., Tomalty D., O’Hara R., Tatum C. (2009). Transarterial chemoembolization of metastatic colorectal carcinoma with drug-eluting beads, irinotecan (DEBIRI): multi-institutional registry. *Journal of Oncology*.

[3] Fiorentini G., Aliberti C., Tilli M. (2012). Intra-arterial infusion of irinotecan-loaded drug-eluting beads (DEBIRI) versus intravenous therapy (FOLFIRI) for hepatic metastases from colorectal cancer: final results of a phase III study. *Anticancer Research*.

[4] Lewis A. L., Holden R. R., Chung S. T. (2013). Feasibility, safety and pharmacokinetic study of hepatic administration of drug-eluting beads loaded with irinotecan (DEBIRI) followed by intravenous administration of irinotecan in a porcine model. *Journal of Materials Science. Materials in Medicine*.

[5] Wang X., Erinjeri J. P., Jia X. (2013). Pattern of retained contrast on immediate postprocedure computed tomography (CT) after particle embolization of liver tumors predicts subsequent treatment response. *CardioVascular and Interventional Radiology*.

[6] Lencioni R., Aliberti C., de Baere T. (2014). Transarterial treatment of colorectal cancer liver metastases with irinotecan-loaded drug-eluting beads: technical recommendations. *Journal of Vascular and Interventional Radiology*.

[7] Ko Y. J., Karanicolas P. J. (2014). Hepatic arterial infusion pump chemotherapy for colorectal liver metastases: an old technology in a new era. *Current Oncology*.

[8] Nicoud I. B., Jones C. M., Pierce J. M. (2007). Warm hepatic ischemia-reperfusion promotes growth of colorectal carcinoma micrometastases in mouse liver via matrix metalloproteinase-9 induction. *Cancer Research*.

[9] Xu G., Zhang W., Ma M. K., McLeod H. L. (2002). Human carboxylesterase 2 is commonly expressed in tumour tissue and is correlated with activation of irinotecan. *Clinical Cancer Research*.

[10] Tobin P., Clarke S., Seale J. P. (2006). The *in vitro* metabolism of irinotecan (CPT‐11) by carboxylesterase and *β*-glucuronidase in human colorectal tumours. *British Journal of Clinical Pharmacology*.

